# Supplementary material for: Mechanistic insight into the functional role of human sinoatrial node conduction pathways and pacemaker compartments heterogeneity: A computer model analysis
Source: PLoS Comput Biol. 2023 Dec 18;19(12):e1011708. doi: 10.1371/journal.pcbi.1011708 (PMC10760897; doi:10.1371/journal.pcbi.1011708)
Supplement: S1 Text — Table A. Relative ratios of the densities of the four key ionic channels (If, INa, IK1 and IKACh currents) among the human SAN head, center and tail, and SACPs used in the computer modeling of the control SAN. SACP- sinoatrial pathways, SAN–sinoatrial node, SCL–sinus cycle length. Table B. Relative ratios of the densities of the four key ionic channels: If, INa, IK1 and IKACh (A1R expression), and fibrosis among the human SAN head, center and tail, and SACPs used in the computer modeling of HF. SACP- sinoatrial pathways, HF–heart failure, A1R –A1 adenosine receptor, SAN–sinoatrial node, SCL–sinus cycle length. Table C. The regional differences in gap junctional coupling between the SAN center, SAN head/tail, SACPs and RA tissues by setting different diffusion coefficients in these regions. SACP- sinoatrial pathways, SAN–sinoatrial node, RA–right atrium. Fig A. 3D microstructural composition of the human SAN complex (an ex-vivo human donor heart,). A, 3D microstructure of all tissue types including myofibers, fibrosis, and fat in the SAN complex (red) and surrounding atrial tissue (green). B, The myofibers of the SAN complex and surrounding atrial tissue. C, The fibrotic fibers of the SAN complex and surrounding atrial tissue. D, The fat texture of the SAN complex and surrounding atrial tissue. SAN–sino-atrial node, CT–crista terminals, IAS–interatrial septum, SVC–superior vena cava, RAA–right atrial appendage. Fig B. The structure of human SAN computer model. The SAN model structure was obtained using a shadow of the 3D SAN reconstruction to the XY plane (parallel to epicardium) by project all 2Ds into one plane. As a result, the 2D representation of the entire 3D human SAN structure included all SACPs and the complete SAN head/center/tail. SAN–sinoatrial node, SACP–sinoatrial pathways. Fig C. Changes in leading pacemaker locations and SACPs in the presence of 20% adenosine due to increasing A1R expression level in the SAN head and tail from 0.1 to 0.9 while keeping [file pcbi.1011708.s001.docx]

**Supplemental Materials**

**Mechanistic insight into the functional role of human sinoatrial node conduction pathways and pacemaker compartments heterogeneity: A computer model analysis**

Jichao Zhao^1*^, Roshan Sharma^1^, Anuradha Kalyanasundaram^2^, James Kennelly^1^, Jieyun Bai^1^, Ning Li^2^, Alexander Panfilov^3^ and Vadim V. Fedorov^2*^

^1^Auckland Bioengineering Institute, University of Auckland, Auckland, New Zealand

^2^Department of Physiology & Cell Biology, Bob and Corrine Frick Center for Heart Failure and Arrhythmia; The Ohio State University Wexner Medical Center, Columbus, OH, United States of America

^3^Gent University, Gent, Belgium

The authors declare no conflicts of interest.

**Address for correspondence:**

Jichao Zhao, PhD

Email: [j.zhao@auckland.ac.nz](mailto:j.zhao@auckland.ac.nz)

Vadim V. Fedorov, PhD

Email: [vadim.fedorov@osumc.edu](mailto:vadim.fedorov@osumc.edu)

# Methods

**Human SAN Optical Mapping and 3D Reconstruction**

Near-infrared otical mapping data and histological imaging and reconstruction of human SAN used for the current SAN model were described and published previously in Li et al. 2017 and 2020[5, 9]. Briefly, an intact cardioplegically-arrested human donor heart without cardiac disease and arrhythmias history was obtained from Lifeline of Ohio Organ Procurement Organization. The Institutional review board defined the study on samples from deceased donors as Not Human Subjects Research. The human SAN preparations were coronary perfused and stained with near-infrared dye di-4-ANBDQBS. The near-infrared transmural optical mapping of the human SAN complex and the entire neighbouring RA was conducted with two MiCAM Ultima-L CMOS cameras (SciMedia, Japan) with a spatial resolution of up to 330 µm^2^. These Intramural near-infrared optical mapping approaches identified the leading pacemakers within the SAN complex, five SACPs and the earliest RA activation sites.

After *ex-vivo* functional mapping, the human SAN preparation was used for 3D structural reconstruction and analysis. Histology sections were imaged at a spatial resolution of 0.5×0.5 µm^2^ using a 20X digital slide scanner (Aperio ScanScope XT, Leica). The high-resolution histology images of the human SAN pacemaker complex were sequentially stacked, and artificial deformation across the z-axis was minimized using a novel 3D image alignment approach[5]. Subsequently, segmentation was performed on the stacks of Masson's trichrome to separate the SAN from the neighbouring RA based on functional and structural data. Myocardial tissue was delineated from fat, blood vessels and fibrosis based on the colour intensity within the 3D SAN complex (**Fig A**).

# Supplementary Table

**Table A**: Relative ratios of the densities of the four key ionic channels (I_f_, I_Na_, I_K1_ and I_KACh_ currents) among the human SAN head, center and tail, and SACPs used in the computer modeling of the control SAN. SACP- sinoatrial pathways, SAN – sinoatrial node, SCL – sinus cycle length.

|  | SAN center | SAN Head/tail | SACP |
| --- | --- | --- | --- |
| I_f_ | 1 | 0.5 | 0.3 |
| I_Na_ | 1 | 2 | 5 |
| I_K1_ | 0 | 0 | 0.2 |
| I_KACh_ | 1 | 0.1 | 1 |
| Fibrosis | 0 | 0 | 0 |
| Single cell SCL | 813 ms | 798 ms | - |

**Table B**: Relative ratios of the densities of the four key ionic channels: I_f_, I_Na_, I_K1_ and I_KACh_ (A1R expression), and fibrosis among the human SAN head, center and tail, and SACPs used in the computer modeling of HF. SACP- sinoatrial pathways, HF – heart failure, A1R – A1 adenosine receptor, SAN – sinoatrial node, SCL – sinus cycle length.

|  | SAN centre | SAN Head/tail | SACPs |
| --- | --- | --- | --- |
| I_f_ | 0.8 | 0.4 | 0.24 |
| I_Na_ | 0.8 | 1.6 | 4 |
| I_K1_ | 0 | 0 | 0.2 |
| I_KACh_/adenosine/A1R | 1 | 0.1 | 1 |
| Fibrosis | 0.2 | 0.2 | 0.2 |
| Single cell SCL | 829 ms | 814 ms | No automaticity |

**Table C**: The regional differences in gap junctional coupling between the SAN center, SAN head/tail, SACPs and RA tissues by setting different diffusion coefficients in these regions. SACP- sinoatrial pathways, SAN – sinoatrial node, RA – right atrium.

| Diffusion coefficient (mm^2^/ms) | SAN center | SAN head/tail | SACPs | RA tissue | Insulating layers of SAN |
| --- | --- | --- | --- | --- | --- |
| Along the fiber | 0.035 | 0.05 | 0.1 | 0.25 | 0.0000025 |
| Across the fiber | 0.0035 | 0.005 | 0.01 | 0.025 | 0.0000025 |

# Supplementary Figures


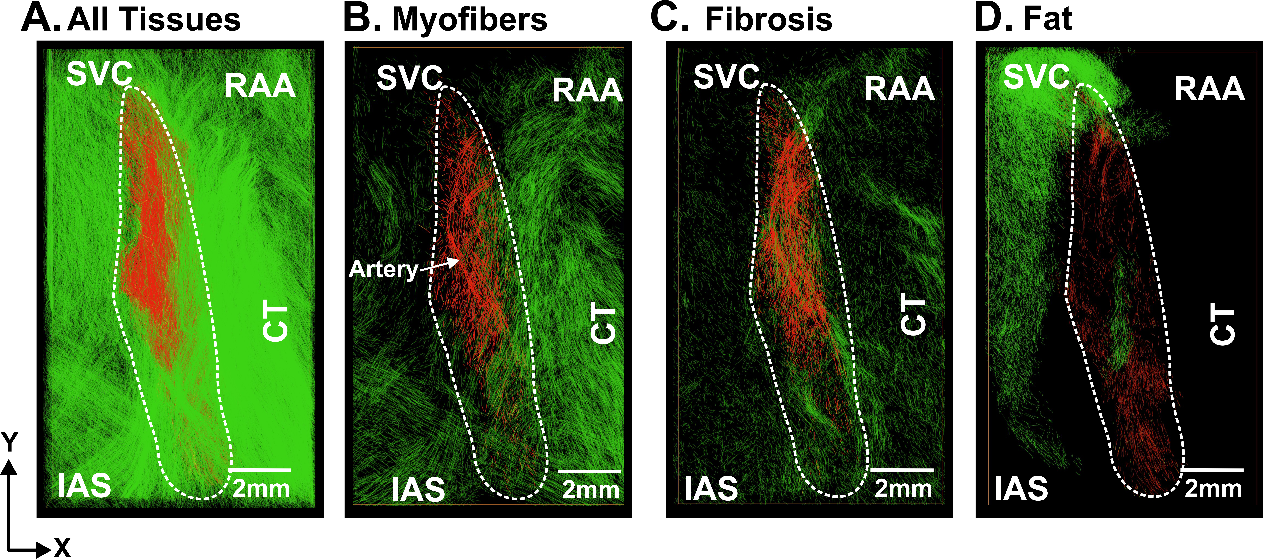


**Fig A**. **3D** **microstructural composition of the human SAN complex** (an *ex-vivo* human donor heart). **A,** 3D microstructure of all tissue types including myofibers, fibrosis, and fat in the SAN complex (red) and surrounding atrial tissue (green). **B,** The myofibers of the SAN complex and surrounding atrial tissue. **C,** The fibrotic fibers of the SAN complex and surrounding atrial tissue. **D,** The fat texture of the SAN complex and surrounding atrial tissue. SAN – sino-atrial node, CT – crista terminals, IAS – interatrial septum, SVC – superior vena cava, RAA – right atrial appendage.


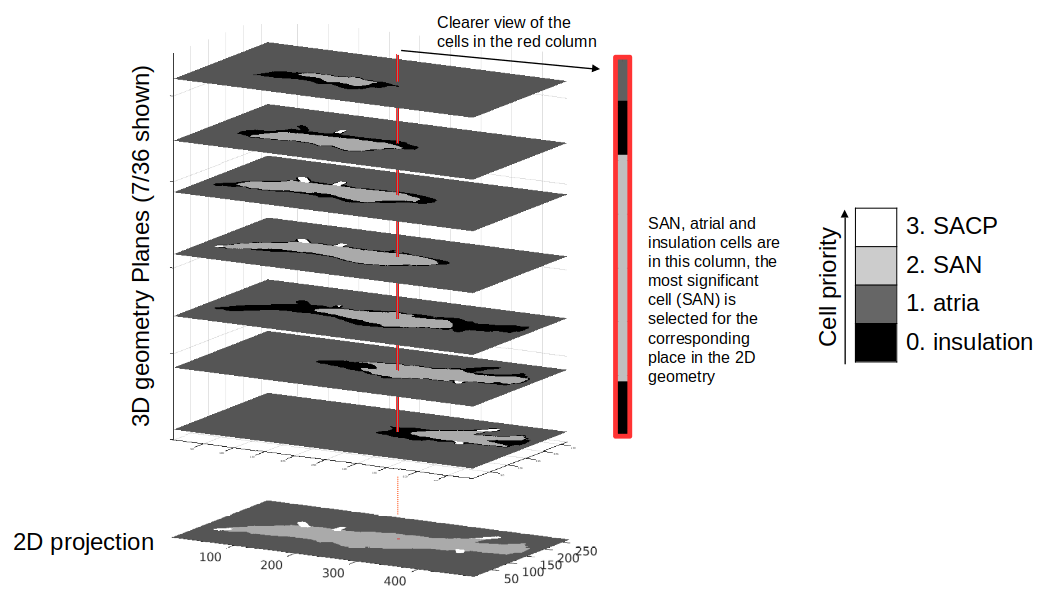


**Fig B**. **The structure of human SAN computer model.** The SAN model structure was obtained using a shadow of the 3D SAN reconstruction to the XY plane (parallel to epicardium) by project all 2Ds into one plane. As a result, the 2D representation of the entire 3D human SAN structure included all SACPs and the complete SAN head/center/tail. SAN – sinoatrial node, SACP – sinoatrial pathways.


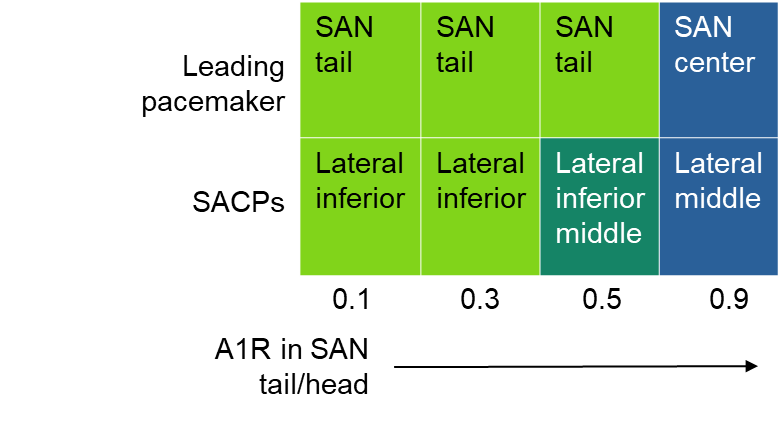


**Fig C. Changes in leading pacemaker locations and SACPs in the presence of 20% adenosine due to increasing A1R expression level in the SAN head and tail from 0.1 to 0.9 while keeping A1R in the SAN center constant as 1**. SACP- sinoatrial pathways, SAN – sinoatrial node, RA – right atrium.


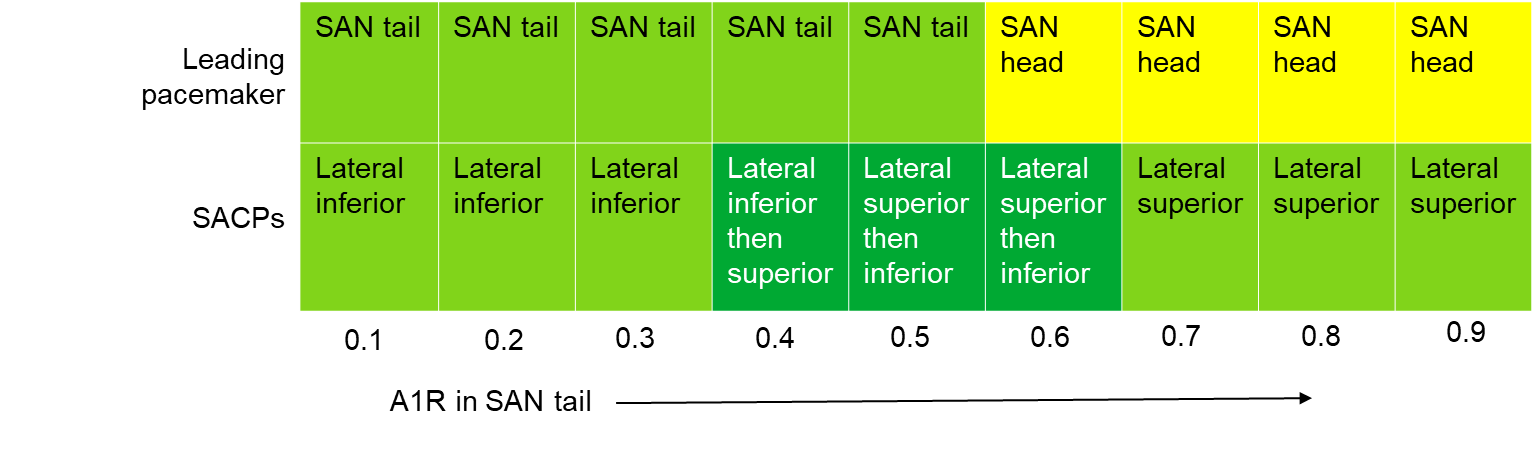


**Fig D. Changes in leading pacemaker locations and SACPs in the presence of 20% adenosine due to increasing A1R expression level in the SAN tail from 0.1 to 0.9 while keeping A1R constant in the SAN head and in the center**. A1R was set at 1 in the SAN center and 0.1 in the SAN head. SACP- sinoatrial pathways, SAN – sinoatrial node, RA – right atrium.


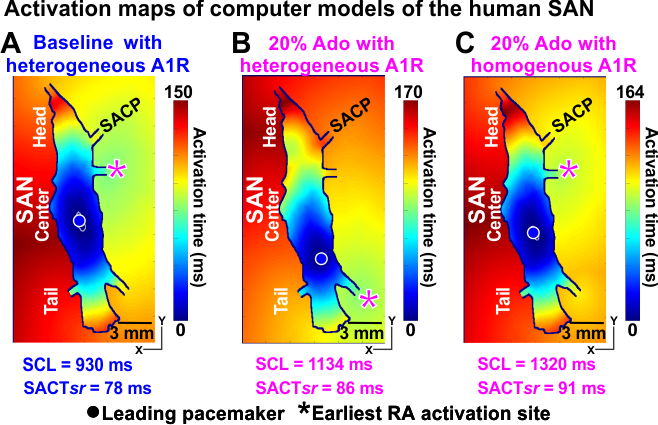


**Fig E**. **The heterogeneity of adenosine A1 receptors (A1R) expressions or the I_KACh_ channel within the intranodal pacemaker compartments (head, center and tail) of the SAN complex is required for the shift in the leading pacemaker and the earliest atrial activation sites during adenosine**. **A,** SAN and atrial activation maps at Baseline. **B,** In the model with heterogeneous A1R/I_KACh_ expression in the SAN center vs head/tail (1.0:0.1), administration of 20% Adenosine led to both the leading pacemaker shift (from center to tail) and the shift of the earliest atrial activation site/ preferential SACP from the lateral to inferior SACP. **C,** In contrast, in the SAN model with homogeneous A1R/I_KACh_ expression across all three SAN compartments (1:1:1), the same 20% Adenosine did not lead to the pacemaker or SACP shift but more severely suppressed SAN automaticity and conduction. The activation maps were almost identical for homogenous A1R with 20% Ado and baseline. SAN – sinoatrial node, SACTsr – SAN conduction time during sinus rhythm*,* SCL – sinus cycle length.

**
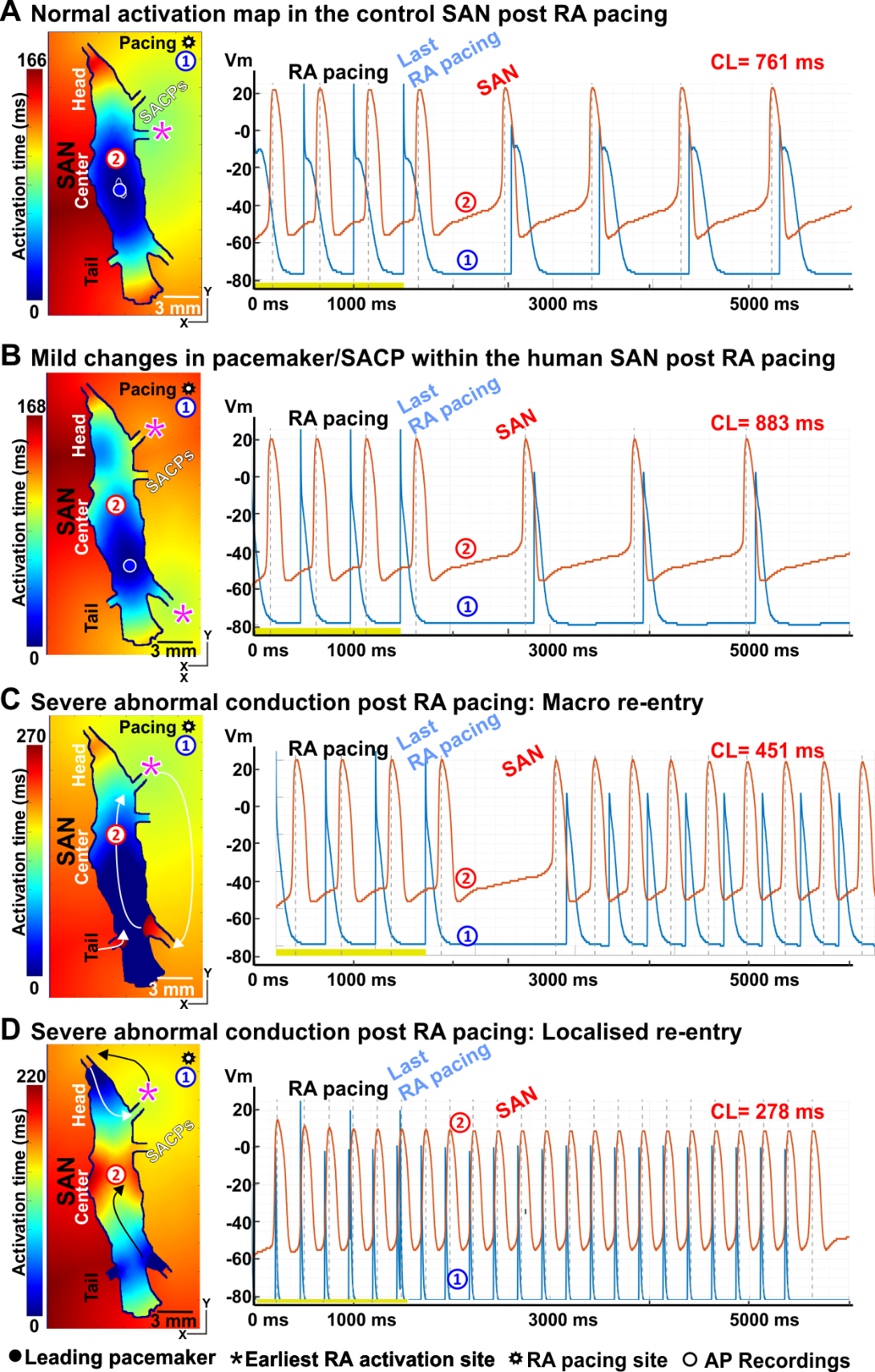
**

**Fig F**. **Four activation patterns were observed after the cession of right atrial (RA) pacing with a CL of 500 ms in the control SAN model** (see **Figure 8B** in the main manuscript). **A,** Normal SAN activation pattern: the first post-pacing SAN beat had the same activation pattern as SAN beats before pacing with the leading pacemaker in the center and preferential conduction exit through the middle lateral SACP. **B,** Mild changes in pacemaker/SACP post the RA pacing: the first post-pacing SAN beat had different activation compared with pre-pacing SAN activation with both leading pacemaker and SACP exit site shifts. **C,** Severe abnormal conduction: SAN macro reentries with slower intranodal conduction path between inferior and superior SACP and a CL of 451 ms spontaneously occurred after RA pacing. **D,** Severe abnormal conduction – Localized SAN reentry between two superior SACPs induced by RA pacing. Two action potential (AP) tracings are from RA, near the pacing site, and the other is located in the center of the SAN*.* SAN – sino-atrial node, SACP – SAN conduction pathway, Ado – Adenosine, AP – Action potential, CL – cycle length.

**
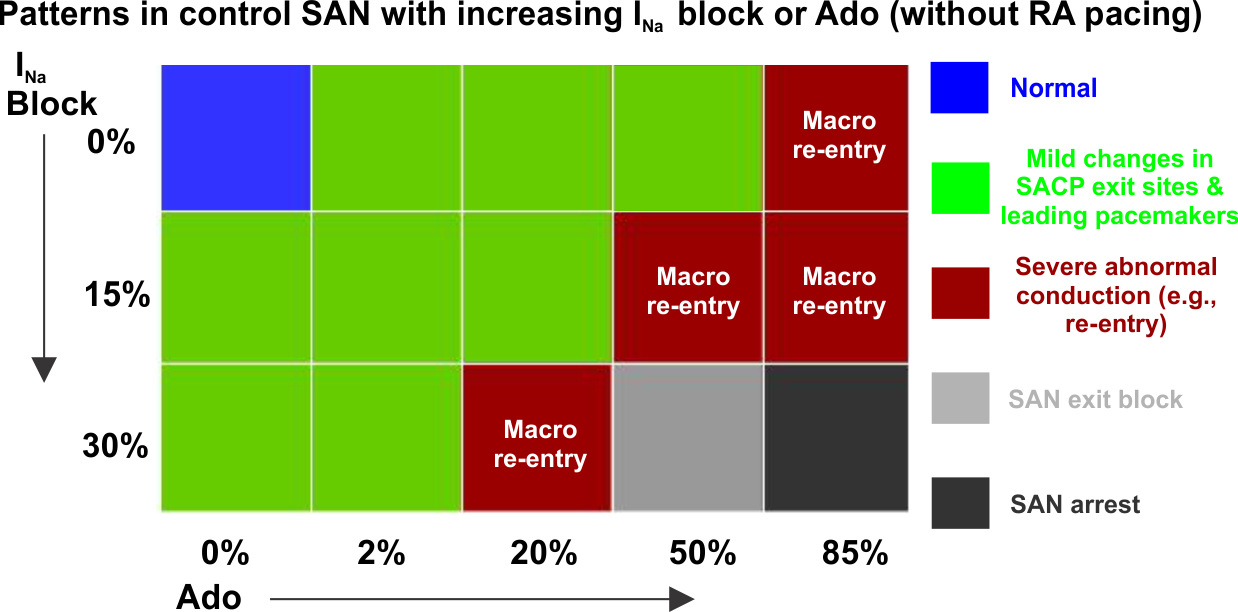
**

**Fig G**. **Summary of propagation patterns in control SAN model with increasing I_Na_ block or Ado** (without extrastimuli). Here Normal (blue square) refers to the leading pacemaker in the SAN center and consistent exit through the lateral middle SACP. SAN – sino-atrial node, SACP – SAN conduction pathway, Ado – Adenosine*.*
