# Supplementary material for: Mechanistic insight into the functional role of human sinoatrial node conduction pathways and pacemaker compartments heterogeneity: A computer model analysis
Source: PLoS Comput Biol. 2023 Dec 18;19(12):e1011708. doi: 10.1371/journal.pcbi.1011708 (PMC10760897; doi:10.1371/journal.pcbi.1011708)
Supplement: Attachment — Submitted filename: RebuttalLetter_2023-03-23.pdf [file pcbi.1011708.s002.pdf]

Dear Dr. Zhao,

Thank you very much for submitting your manuscript "Mechanistic insight into the functional role of human sinoatrial node conduction pathways and pacemaker compartments heterogeneity: A computer model analysis" for consideration at PLOS Computational Biology.

As with all papers reviewed by the journal, your manuscript was reviewed by members of the editorial board and by several independent reviewers. In light of the reviews (below this email), we would like to invite the resubmission of a significantly-revised version that takes into account the reviewers' comments.

The paper is of interest, but in its current form the level of detail provided on model creation and validation are insufficient, so the quality of the paper can not be evaluated.

Please make sure that the data sources used to create and validate the model are referenced and that the species and temperature of any data sources are clearly stated.

Please also make sure that model code is available. Ideally submitted to a repository (cellml for example).

We cannot make any decision about publication until we have seen the revised manuscript and your response to the reviewers' comments. Your revised manuscript is also likely to be sent to reviewers for further evaluation.

When you are ready to resubmit, please upload the following:

[1] A letter containing a detailed list of your responses to the review comments and a description of the changes you have made in the manuscript. Please note while forming your response, if your article is accepted, you may have the opportunity to make the peer review history publicly available. The record will include editor decision letters (with reviews) and your responses to reviewer comments. If eligible, we will contact you to opt in or out.

[2] Two versions of the revised manuscript: one with either highlights or tracked changes denoting where the text has been changed; the other a clean version (uploaded as the manuscript file).

Important additional instructions are given below your reviewer comments.

Please prepare and submit your revised manuscript within 60 days. If you anticipate any delay, please let us know the expected resubmission date by replying to this email. Please note that revised manuscripts received after the 60-day due date may require evaluation and peer review similar to newly submitted manuscripts.

Thank you again for your submission. We hope that our editorial process has been constructive so far, and we welcome your feedback at any time. Please don't hesitate to contact us if you have any questions or comments.

Sincerely,

Steven A. Niederer
Guest Editor

PLOS Computational Biology

Daniel Beard
Section Editor
PLOS Computational Biology

****

The paper is of interest, but in its current form the level of detail provided on model creation and validation are insufficient, so the quality of the paper can not be evaluated.

Please make sure that the data sources used to create and validate the model are referenced and that the species and temperature of any data sources are clearly stated

Please also make sure that model code is available. Ideally submitted to a repository (cellml for example).

Response: We would like to thank the editor for the detailed suggestions and apologize for not explaining the two cellular models we used in this study more explicitly.

- The human SAN cellular model was based on the Fabbri et al. J Physiol. 2017, which was in the CellML depository (please refer to DOI:10.36903/physiome.16550526 or Physiome repository: https://models.physiomeproject.org/workspace/648)
- The human atrial cellular model was based Ni et al.,. *Front Physiol.* 2017 (please refer to https://models.physiomeproject.org/e/807/ni_2017.cellml/view)

As you can see these two original cellular models were already in the CellML depository, and in their original journal articles, the authors have provided details of the data sources of species and temperature. Therefore, we believe that there is no need to elaborate more in this study since we did not create any cellular model ourselves in this study.

In our study, we applied and adapted (very modestly) the original cellular SAN models for different SAN regions with SAN regional heterogeneity (Online Tables S1&S2) to qualitatively match published experimental results (Li et al., 2017 & 2020) to investigate mechanisms of SAN automaticity and conduction, and SAN arrhythmia mechanisms under different assumptions. A custom-built C software was used to generate all computer simulations and has been described previously by our group (Zhao et al. Circulation: Arrhythmia and Electrophysiology 2012, 5 (2), 361-370) and others (Seemann and Zhang et al. 2006 https://doi.org/10.1098/rsta.2006.1781).

In our revision, custom C code and related files used for computer simulations and the human SAN model in this study have been deposited at GitHub: https://github.com/rsha919/Human_SAN_2D_Fabbri_Ni.

At the end of the revised manuscript, we have added a new subsection for code availability:

"Code availability
The two original human SAN[16] and RA[22] cellular models we used in this study are in the CellML depository. The human SAN cellular model can be downloaded from Physiome CellML repository: https://models.physiomeproject.org/workspace/648. The human atrial cellular model is accessible through https://models.physiomeproject.org/e/807/ni_2017.cellml/view. In our study, we applied and adapted the original cellular SAN models for different SAN regions with SAN regional heterogeneity to qualitatively match experimental results. A custom-built C software was used to

generate all computer simulations and has been described previously by our group[9,15]. Custom C code and related files used for computer simulations and the human SAN model in this study have been deposited at GitHub: https://github.com/rsha919/Human_SAN_2D_Fabbri_Ni."

Responses to Reviewer's Questions

Comments to the Authors:
Please note here if the review is uploaded as an attachment.

Reviewer #1: *The paper presents a detailed biophysical modelling study of pacemaking in the human sinoatrial node (SAN). The 2D SAN models are based on a mixture of experimental structure-functional data collected by the authors in previous studies and existing electrophysiology models and parameters from literature. Various pacemaking conditions are considered, including adenosine modulation, alterations of ionic currents, leading pacemaker shift, fibrotic remodelling, electrical insulation of the SAN and heaty failure. Strengths of the paper include the high level of detail in the model, it's good agreement with a range of experimental observations and mechanistic explanations of some aspects of the human pacemaking. Weaknesses include a lack of clarity in explaining the underlying methods, in systemizing/generalizing the numerous modelling results and in delineating the novelty and importance of specific conclusions.*

Response: We would like to thank Dr. Oleg Aslanidi for the positive endorsement, detailed suggestions and comments, which were used to improve our revised manuscript significantly.

METHODS

"Ex-vivo Human SAN and Optical Mapping" and "Histological Imaging and Reconstruction of Human SAN" sections - please clarify what has been done in your previous studies, and what was done specifically for the current study; also clarify what types of data used in the models were 'heart-specific' and what types were generic (e.g., taken from previous experimental or modelling studies).

Response: We apologize for not explaining more explicitly as to what was done in our previous studies and what was new in the current study.

For the specific human heart (38 y.o. male) used in the current computer modeling study, the near infrared optical mapping, 3D histological imaging and detailed analysis were published by Li et al. *Science Translational Medicine* 2017, while the specific structural analysis of the human SAN reconstruction was published by Csepe et al. *Prog Biophys Mol Biol.* 2016. In this study, we set up and conducted the computer models of the human SAN complex for electrical simulation to investigate mechanisms of SAN automaticity and conduction, and SAN arrhythmia mechanisms under different pathophysiological conditions.

Additionally, in our computer simulations, the modeling anatomy of the human SAN and SACPs were heart-specific, while cardiac electrophysiology in the RA and SAN under normal and different pathophysiological conditions were generic, which were based on previous experimental or modeling studies.

"The optically mapped 3D SAN activation maps were used to guide SAN histological dissection (Figure 1A)" – from Fig. 1 is it absolutely unclear how 3D activation maps were used.

Response: Our apologies for the confusion and we agree with the Reviewer's comment. The detailed approach was explained in Csepe et al. *Prog Biophys Mol Biol.* 2016. We have now added this reference at the end of this sentence and removed Figure 1A:

"After ex-vivo functional mapping, the human SAN preparation was used for 3D structural reconstruction and analysis. Intramural SAN and atrial surface activation maps, immunostaning and histological imaging were used to identify the SAN pacemaker conduction complex.[5]"

"Subsequently, a semi-manual segmentation was performed on the stacks of Masson's trichrome" – please explain what is meant by 'semi-manual', e.g. what steps were not manual and how exactly they were performed.

Response: We have a detailed description of the segmentation approach in Csepe et al., *Prog Biophys Mol Biol.* 2016 (please see below). This new study focuses on computer modeling of the human SAN complex. To avoid confusion, we have removed "semi-manual" in our revised manuscript, and referred to the detailed approach in Csepe et al., Prog Biophys Mol Biol. 2016.

Csepe et al., Prog Biophys Mol Biol. 2016: "High-resolution histology images of human right atria and the SAN pacemaker complex were sequentially stacked and artificial deformation across the z-axis was minimized using a novel 3D image alignment approach by applying a global elastic constraint via an ImageJ Plugin (TrakEM2) (Figure 4D)[42]. The package automatically aligns a series of registered RGB images by employing a suite of tools including an affine transformation and least squares (linear) to find effective feature correspondences among the neighboring original images[42] in order to accurately align and combine 2D images for subsequent 3D reconstruction and analysis (Figure 4E). Then a semi-manual segmentation was performed on the 2D stacks of Masson's trichrome images with 0.5×0.5 μm2 in-plane and 21 μm (Heart #1) or 13μm (Heart #2) across-plane resolution to separate the SAN from neighboring atrial tissue based on functional and structural data (Figure 4A-C). The resulting 3D surface was further incorporated and smoothed through commercial software Amira (FEI, Oregon, USA) (Figure 4E). High-resolution fiber fields were obtained using Eigen-analysis of the 3D structure tensor. The 4D myofiber field was further computed using a state-of-art fiber tracking approach by seeding uniformly throughout a region of interest via a custom-developed Matlab package (MathWorks Inc., Natick, Massachusetts, USA) and visualized by commercial software Amira[43, 44]. The fiber tracking approach utilized a linear line propagation algorithm i.e., a single fiber line propagated by connecting pixels from a seed point by following the local vector orientation until it reaches beyond tissue region or predefined angle change[44]..."

"The resultant SAN computer model was obtained by projecting the 3D SAN modeto the XY plane" – it's unclear what is meant by 'projecting', why you didn't you simply use the 2D slices seen in Fig. 1B?

Response: The Reviewer is correct, the wording 'projecting' that we used here was not accurate. Probably, 'Shadow' is a better word. Basically, what we need here is a shadow of the 3D human SAN, i.e., a 2D reconstruction of 3D human SAN to include all SACPs and the complete SAN head/center/tail, instead of the only one single 2D slice seen in Fig. 1. As any single 2D slice across 3D human SAN pacemaker complex could not include all five SACPs.

In the revised manuscript, we changed accordingly: "*The SAN computer model was obtained using a shadow of the 3D SAN model to the XY plane (parallel to epicardium). As a result, the 2D representation of the entire 3D human SAN structure included all SACPs and the complete SAN head/center/tail, which is crucial for the aims of this study."*

"The insulating wall (at a uniform thickness of two pixels) between the SAN and RA was given a constant potential of -62.5 mV" – where did this value was taken from?

Response: We apologize for the lack of clarity. Our main aim here was to reproduce the insulation of the SAN from atria in the most simple and efficient computational way. For that, any constant value for the insulating wall between the resting potential of the human SAN (between −30 and −50 mV**)** and RA (between −65 and −80 mV) cells will do the work. In our study, we used a value of -62.5 mV, which in addition to insulation has only very slight depolarization and hyperpolarization effects on neighbouring RA and SAN cells, respectively which is more realistic. In general, due to the limited spatial effects of boundary conditions we believe that our results and main conclusions are not affected by the exact choice of that value of constant potential.

In the revised manuscript, we changed accordingly: "*The insulating wall (at a uniform thickness of two pixels) between the SAN and RA was given a constant potential between the resting potentials of the human SAN and RA cells, and a neglectable diffusivity."*

"…we considered the regional differences in gap junctional coupling between the SAN center, SAN head/tail, SACPs and RA tissues by setting the diffusion coefficients at a ratio of 2:3:6:14 in these regions. In the models, an anisotropic diffusivity ratio of 1:10 was used" – what were the actual (absolute) values of the diffusion coefficients?

Response: In the revised manuscript, we have provided the values in Supplementary Table S3.

RESULTS

Figures 2 and 3 – right panel in Fig. 2A and right panel in Fig. 3A both appear to show the same simulation (Ado 85%). However, in Fig. 2 this panel is match up with Ado 10um experimental data (where the leading pacemaker is shifted to the tail), whereas in Fig. 3 it is match up with Ado 100 um experimental data (where the leading pacemaker is shifted to the head most of the time) – with different conclusions drawn from these comparisons to the same Ado 85% simulation. Please used Ado 85% simulations consistently and make consistent conclusions.

Response: We appreciate this specific comment and agree that our original Figs. 2&3 could be confusing. In our revised Fig. 3A, we have replaced the rightmost subfigure 85% Ado by 100% Ado. As such, our computer modeling results using one specific human SAN model indicated that under baseline conditions in the computer model (Fig. 2A left), the leading pacemaker was located in the SAN center (circle), and the earliest RA activation site was through the middle lateral SACP. In the presence of Ado, such as 50% or 80% of the maximum dose, the leading pacemaker shifted to the SAN tail while the earliest RA activation site was activated through the inferior (50% Ado) and superior (80% Ado) lateral SACPs, respectively. At 100% Ado, it led to cardiac arrest.

We have also substantially rewritten our manuscript to clarify the relation between computational and modelling results and we now clearly indicate which experimental observations are consistent with our modelling results: *"Experimental results in the optically mapped human hearts ex-vivo (n=11) at baseline and in the presence of low (10 μM) and high (100 μM) concentrations of adenosine are shown in **Figure 3B**. In the functionally mapped explanted human SANs (n=11), the increasing dose of adenosine led to a heart-specific pacemaker shift toward the head or tail of the SAN complex. An increase in concentrations of adenosine led to a higher chance of*

conducting via superior or inferior lateral SACPs as in computer model. Also, 100 μM Ado led to cardiac or SAN arrest in five out of the 11 hearts similar to what we observed in our simulations"

Our revised Figure 3:

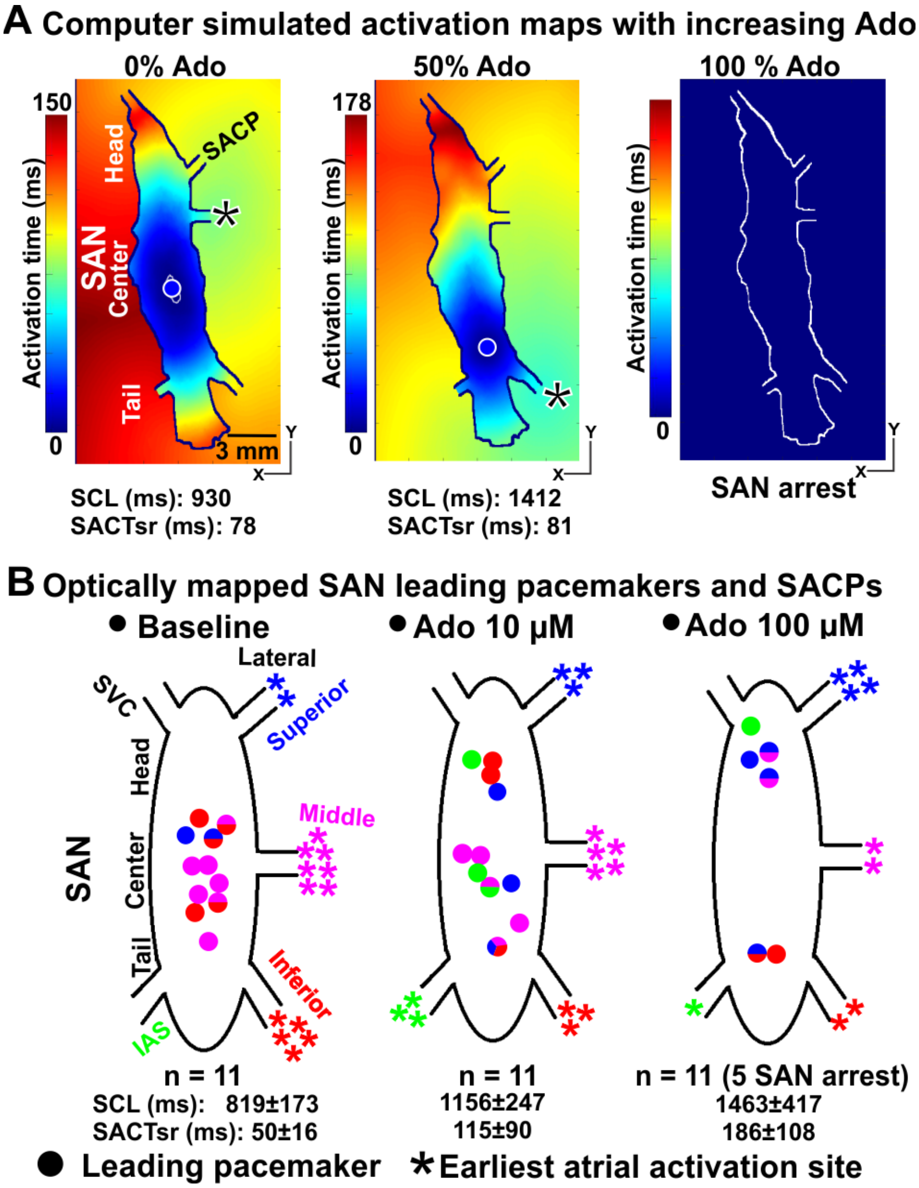

***Figure 3**. **A shift in the leading pacemaker and earliest atrial activation sites in the human SAN model with varying Ado concentration**. **A,** Increasing the presence of Ado (from 0% to 100%) in the computer model of the human SAN complex led to a shift in the leading pacemaker and earliest atrial activation sites, eventually exit block and complete atrial arrest (100% of Ado). **B,** A similar shift in the leading pacemaker and earliest atrial activation sites was observed in 11 optically mapped human hearts ex-vivo in the absence and presence of Ado.[1] The increasing dose of Ado led to a heart-specific pacemaker shift toward the head or tail of the SAN complex, and a higher chance of conducting via superior or inferior lateral SACPs as in computer model. Also, 100 μM Ado led to cardiac or SAN arrest in five out of the 11 hearts. SAN – sino-atrial node,*

SCL – sinus cycle length, SACP – sino-atrial conduction pathway, SACTsr – sinoatrial conduction *time during sinus rhythm*, *Ado – Adenosine.*

One puzzling observation in Fig. 3 – in Ado 100 um experiment the leading pacemaker is shifted to the SAN head most of the time, and it makes sense that the earliest atrial activation is near there in the head; however, in the matching Ado 85% simulation the leading pacemaker is shifted to the tail, but the earliest activation site is still in the head, through the furthermost SACP. Any explanation?
In the light of my comments above, please rephrase the sentence "Somewhat similar trends were observed in the optically mapped human hearts ex-vivo at baseline and in the presence of low (10 μM) and high (100 μM) concentrations of adenosine (Figure 3B)."

Response: We appreciate this very insightful question. We have completely re-written the text on the relation of computational and experimental results presented in Fig.3. *"Importantly, increasing the concentration of adenosine from 0% to 100% in the computer models (**Figures 2A&3A**) led to a progressive slowing of the SCL and SACT in parallel with shift in the leading pacemaker and earliest atrial activation sites, until complete SAN arrest at 100% adenosine concentration. The simulation results show that the leading pacemaker shifted inferiorly from the SAN center to the SAN tail l, while the earliest RA activation site first shifted to the inferior lateral SACP for adenosine concentration 50% (Figure 3A) and then to superior lateral SACP at adenosine concentration 85% (Figure 2A). Both SCL and SACTsr were increased in the computer model with an increasing dose of adenosine. Experimental results in the optically mapped human hearts ex-vivo (n=11) at baseline and in the presence of low (10 μM) and high (100 μM) concentrations of adenosine are shown in **Figure 3B**. In the functionally mapped explanted human SANs (n=11), the increasing dose of adenosine led to a heart-specific pacemaker shift toward the head or tail of the SAN complex. An increase in concentrations of adenosine led to a higher chance of conducting via superior or inferior lateral SACPs as in the computer model. Also, 100 μM Ado led to cardiac or SAN arrest in five out of the 11 hearts similar to what we observed in our simulations."*

The Reviewer asked whether the earliest RA activation is not always via the closest SACP to the leading pacemaker is unique to our computer simulations. We have often observed this pattern in our optical mapping of the human SANs. For instance, in Fig. 3B, two of the six leading pacemakers, i.e., the topmost and left-bottom pacemakers, led to the earliest RA activation via distal SACPs (not the closest SACP) (Li et al. *Science Translational Medicine* 2017; Li et al. *Nature Communications* 2020). The mechanism underlying this can be complex. One crucial factor is that sharp myofiber orientation changes in the junction of the SAN and SCAPs may favour one over others (Spach and Bioneau, *PACE* 1997).

Figure 4 – quite a few heterogeneity parameters are being varied, it could be better to organize numerical data into a summary figure (similar to how it's done in Fig. 7) or a table, rather than write multiple numbers on top the figures; to a lesser extent, this might also be beneficial for Figs. 5-7.

Response: We appreciate these constructive comments from the Reviewer. However, we did not run the computer simulations for all the value A1R ranges of SAN tail/head/center since it is exceptionally time-consuming for a biophysics-based computer model. Based on the available simulation data (current Figures 4C&D), we have made two new tables in the revised

Supplemental Materials as suggested by the Reviewer. The new Supplemental Table S4 (see below) is a summary of Figure 4C, showing changes in leading pacemaker locations and SACPs leading to the earliest RA activation with increasing A1R in the SAN head/tail. The new Supplemental Table S5 (see below) is a summary of Figure 4D, showing changes in leading pacemaker locations and SACPs leading to earliest RA activation with increasing A1R in the SAN tail only.

Supplementary Table S4: It shows changes in leading pacemaker locations and SACPs leading to the earliest RA activation with increasing A1R in the SAN head and tail while keeping the SAN center constant as 1 (in the presence of 20% adenosine). SACP – sinoatrial pathways, SAN – sinoatrial node, RA – right atrium.

Leading pacemaker	SAN tail	SAN tail	SAN tail	SAN center
SACPs	Lateral inferior	Lateral inferior	Lateral inferior middle	Lateral middle
	0.1	0.3	0.5	0.9

A1R in SAN tail/head →

Supplementary Table S5: It shows changes in leading pacemaker locations and SACPs leading to the earliest RA activation with increasing A1R in the SAN tail while keeping SAN head and center constant (in the presence of 20% adenosine). A1R was set at 1 to the SAN center and 0.1 to the SAN head. SACP- sinoatrial pathways, SAN – sinoatrial node, RA – right atrium.

Leading pacemaker	SAN tail	SAN tail	SAN tail	SAN tail	SAN tail	SAN head	SAN head	SAN head	SAN head
SACPs	Lateral inferior	Lateral inferior	Lateral inferior	Lateral inferior then superior	Lateral superior then inferior	Lateral superior then inferior	Lateral superior	Lateral superior	Lateral superior
	0.1	0.2	0.3	0.4	0.5	0.6	0.7	0.8	0.9

A1R in SAN tail →

The key message in Figure 6 was to show the shifting in the leading pacemaker, automaticity and earliest atrial activation sites in the presence or absence of the insulation boundary between the SAN and the lateral RA, as well as the critical protective role of the insulation boundary to Adenosine inhibitory effects. This information is difficult to quantify and present in numerical data. We will work on a potential approach to do such in a future study.

For Figures 5&7, we do not believe that new summary figures/tables will help to convey the key messages from the results. The key message from Figure 5A&B is that a small perturbation (+/-15%) in the I_{Na} current in SACPs can influence the earliest atrial activation sites. In contrast, the perturbations of I_f current only influence SCL even though we changed it in **all** SAN compartments and SACPs. The key message from Figure 7B-D is that HF ionic channel remodeling influenced the SCL, earliest atrial activation sites and increased chances of cardiac arrest, while fibrotic remodeling in SACPs increased the chance of SAN exit block.

Figure 7 – it's unclear whether fibrotic remodelling was applied (and then reversed) in the surrounding atrial myocardium, or the SACPs only? In the latter case, why it wasn't applied in the entire atrial tissue?

Response: Indeed, fibrotic remodelling was applied to the SAN and/or SACPs, and then reversed in the SACPs only. This study focused on investigating mechanisms of SAN automaticity and conduction, as well as SAN arrhythmia mechanisms. As such, we did not include fibrotic remodelling in the RA to make it easier to compare computer simulation results between different scenarios within the human SAN complex. We agree that atrial fibrosis around SAN complex may also influence SAN function and we will consider this in future studies.

Figure 8, "In the computer model of the human SAN complex, a train of stimuli at a pacing cycle length of 500 ms was delivered from the right superior RA" – why this specific pacing site and this specific rate were chosen? Would the results be different if a different pacing setting is chosen? It might be necessary to perform some kind of sensitivity analysis to draw general conclusions in this case study.

Response: In the computer models, we used the same RA pacing site (RA superior) and pacing rated to challenge the robustness of SAN pacemaking and conduction, as we used previously in the ex-vivo optical mapping of the human SAN (Li et al. *Nature Communications* 2020). Within a slight change in the pacing sites or rates, the observed conduction patterns will be qualitatively the same and have no impact on our results and conclusions. Quite different pacing rates (e.g., at a pacing cycle length of 200 ms) or sites (e.g., at the inferior RA) may produce different results. A robust sensitivity analysis of the pacing site/rate is out of the scope of this study. We will consider these variables in our future studies. Nevertheless, the focus of this study was to utilize computer modelling consistent with current experimental studies to provide a more systematic analysis of propagation patterns in control and HF conditions with varying I_{Na} block and Ado concentrations (Fig. 8B &8C), and in the absence of RA pacing.

DISCUSSION

"The heterogeneity in expression of adenosine A1 receptors (A1R) or the IKACh channels within the human SAN pacemaker compartments explains leading pacemaker and preferential SACP

shifts in the presence of adenosine" – this conclusion is very general, can it be broken down into more specific conclusions? In regard to the leading pacemaker, please also see my first set of comments on Results.

Response: The Reviewer is right. But here under the Discussion, we want to have a high-level summary of our key results in this study. The novelty of this computer modeling study (over the previously published experimental observations) is to provide direct evidence that the heterogeneity in expression of adenosine A1R or the I_{KACh} channels within the human SAN pacemaker compartments explains leading pacemaker and preferential SACP shifts in the presence of adenosine. For more specific results, we have provided detailed observations under the Results section (after the heading "*The heterogeneity in expression of A1 adenosine receptors or I_{KACh} channel explains pacemaker shifting*").

"The electrical insulation boundary between the SAN and RA except the SACP is required for normal SAN pacemaker and conduction function and to reproduce the leading pacemaker and the earliest atrial activation sites, observed in experimental and clinical studies" – this is one of the most important conclusions, please cite relevant experimental and clinical studies, either here or in Results.

Response: Originally, we aimed to have this high-level summary of our key results at the beginning of the Conclusions, thus we did not cite the references here. Prompted by the Reviewer, we added three studies (Li et al. *Science Translational Medicine* 2017; Li et al. *Nature Communications* 2020; Kalyanasundaram et al., Heart Rhythm 2022) in the revised Results section under The Role of the Insulation Boundary between the SAN and RA.

"The INa current density and fibrotic remodelling (e.g. in heart failure HF) in SACPs modulate the SAN conduction (e.g. exit block) and the preferential SACP/exits to the atria (e.g. earliest atrial activation)" – this conclusion is interesting in the SAN context, but not at all surprising, since both blocking INa and increasing fibrosis are known to slow down or halt conduction. It's worth at least referring to other (mechanistic) studies that explain this, e.g. for the SAN or/and for the atria.

Response: We agree with the Reviewer for his comment that the reduction in I_{Na} current density and fibrotic remodelling slow down conduction is widely accepted and is a well-known arrhythmogenic factor (Refs 27-30). Thus we followed the Reviewer's suggestion and added references which show similar results in the revised manuscript. But the novelty of this study is that the I_{Na} current density and fibrotic remodelling in **SACPs** are more important than in other SAN regions. This is a new finding to our knowledge.

References:

27. King, James H., Christopher L-H. Huang, and James A. Fraser. "Determinants of myocardial conduction velocity: implications for arrhythmogenesis." *Frontiers in physiology* 4 (2013): 154.

28. Morgan, R., Colman, M., Kruger, M., Seemann, G., Rhode, K., & Aslanidi, O. (2014, September). Evaluating effects of fibrosis in atrial arrhythmogenesis using 3D computational modelling. In *Computing in Cardiology 2014* (pp. 765-768). IEEE.

29. Roy, Aditi, Marta Varela, and Oleg Aslanidi. "Image-based computational evaluation of the effects of atrial wall thickness and fibrosis on re-entrant drivers for atrial fibrillation." *Frontiers in physiology* 9 (2018): 1352.

30. Rivaud, Mathilde R., Mario Delmar, and Carol Ann Remme. "Heritable arrhythmia syndromes associated with abnormal cardiac sodium channel function: ionic and non-ionic mechanisms." *Cardiovascular research* 116, no. 9 (2020): 1557-1570.

Under the revised Discussion section, we added *"The reduction in I_{Na} current density and fibrotic remodelling slow down conduction is widely accepted and are well-known arrhythmogenic factors.[27-30] However, the novelty of this study is that the I_{Na} current density and fibrotic remodelling in SACPs are more important than in other SAN regions. This is a new finding to our knowledge."*

"Human SAN pacemaker-conduction complex is vulnerable to reentrant arrhythmia and exit block under the mild intranodal INa current suppression and low-dose adenosine or HF-induced functional and structural remodeling" – again, this is quite general and may be worth breaking down into more specific conclusions. Besides, even without HF the SAN is prone to reentrant arrhythmia (Figure 8B), so it's beneficial to explain how exactly HF makes the situation even worse (in a clinical sense).

Response: We appreciate this comment from the Reviewer. We changed this in the revised manuscript *"Intranodal I_{Na} current suppression or low-dose adenosine intervention leads to shifts in the leading pacemaker and the earliest atrial activation sites and renders the human SAN pacemaker-conduction complex vulnerable to SAN-RA reentry, SAN exit block and arrest. The SAN HF model had a higher incidence of exit block regardless of the presence or absence of I_{Na} block or adenosine."*

"Critical Insights Learned and Clinical Implications" section – almost this entire section talks about pacemaking mechanisms, rather than clinical implications. Only the very last two sentences have some clinical relevance, but even they provide no specific clinical insights. I suggest to rename this section.

Response: We agree and removed "and Clinical Implications" in the revised manuscript.

Reviewer #2: This paper presents a computer model of the sinoatrial node based on histological data. Behaviour is compared with biological experiments. Based on the model, several conclusions are made, including the effects of heart failure, and SAN insulation. The SAN preparation and optical mapping are truly at the forefront of the field. The model proposed is quite complex, and could be of great potential use, but some of the assumptions need better justification, putting the conclusions in doubt. Sensitivity analyses should be performed to gauge the importance of parameters which have been estimated. The paper is well written and argued, otherwise. Detailed comments follow.

Response: We thank the Reviewer for these positive comments and helpful suggestions. Please see our detailed response to the concerns raised below.

The center of the SAN has a higher intrinsic frequency that the head and the tail, which are treated as being the same. Do the authors have data to support this? Traditionally, a monotonically decreasing intrinsic frequency gradient has been described. The recent study of Brennan et al (JACC Clin Electrophys 2020) supports this notion. How do the ionic distributions used compare to those in the aforementioned paper?

Response: Thank the Review for this important question.

We agree that decreasing intrinsic frequency gradient within the SAN complex was widely supported and reported in animal models. Our previous published human SAN experimental data (Li et al., *STM* 2017 and *Nature Communications* 2020) shows that the presence of the leading pacemaker primarily in the central compartment at baseline conditions (with superior SACP/exits to RA) and pacemaker shift was observed to inferior (tail) or superior (tail) compartments during Adenosine and/ or sodium channels inhibitions.

This modeling study supported this observation qualitatively. To reproduce experimental ex-vivo optical mapping results bymodifying I_f and other currents (see Supplemental Table S1) simulated SCLs of isolated single SAN cells in the SAN center and head/tail were 813 ms and 798 ms, respectively (Fig. 1B). The cellular modifications in our computer models were made by considering exclusively human SAN functional and molecular regional heterogeneity data from recent studies (Li et al. 2015, 2017, 2020; Chandler et al. 2009). Particularly, we showed that three HCN isoforms HCN1, 2 and 4, GIRK1/4 channel, adenosine A1 receptors are expressed differently across SAN head, center and tail pacemaker compartments in a heart-specific pattern (Li et al. 2015, 2017, 2020). While other expression in the human SAN vs RA has been reported by Chandler et al. (Chandler et al. 2009) and Li et al. 2021, it is currently not reported their compartment-specific expression patterns within the intranodal compartments of the human SAN. Currently, we are conducting a comprehensive study of the human SAN compartment- and cell-specific pacemaker-conduction genes expression profiling, which will be used for our next modeling studies.

We apologize for missing the comparisons with *Brennan et al. JACC Clin Electrophys 2020*. Brennan et al. could not find the key SAN pacemaker channels expression, e.g. HCN1 or HCN4 higher expression in human SAN samples vs RA, and reported in Discussion "Tissue-level RNA sequencing data from the human SAN tissues extracted for this study did not identify markedly different levels of pacemaking specific ion channels (e.g., HCN1 or HCN4) or connexins (e.g., Cx40 or Cx43) between either pacemakers or atrial myocardium, indicating significant presence

of remnant atrial tissue present in the nodal tissue samples." Thus, it is difficult to provide direct comparisons between our studies.

The authors do not mention adrenergic effects which counter sympathetic effects but not always directly.

Response: This study aimed to conduct computer simulations to elucidate the role of these structural-molecular factors in the functional robustness of human SAN, based on our experimental mapping of the human hearts. Investigating adenosine effects (we believe the Reviewer asked us about "adenosine effects" rather "adrenenrgic effects"), which counter sympathetic effects is out of the scope of this modeling study.

The authors modelled the insulative layer as a fixed voltage of -62.5 mV. Why not just remove the layer as there will be coupling effects which should not be present if truly isolated.

Response: We agree with the Reviewer on this insightful suggestion. However, in reality, a neighbouring RA tissue to SAN will have a coupling effect with SAN even if it is not actively conducting, such as fat. In our computer simulations, we found the insulating wall with a pre-set potential ensured a slight depolarization and hyperpolarization of neighbouring RA and SAN cells, respectively, leading to more biophysical results than a truly isolated but non-realistic layer.

In Methods Realistic Human SAN Computer Model, what do the authors mean by saying, "the neighboring RA and was much more efficient to run than a computer model of the SAN directly based on the 3D histological data."

Response: We apologize for the confusion. The wording 'projecting' that we used here was not accurate. Probably, 'Shadow' is a better word. Basically, what we need here is a shadow of the 3D human SAN, i.e., a 2D reconstruction of 3D human SAN to include all SACPs and the complete SAN head/center/tail, instead of the only one single 2D slice seen in Fig. 1B. A computer model of the 2D SAN is much more efficient than a 3D SAN model directly based on the 3D histological data while keeping essential SAN structural factors in the models.

How do the authors justify the quantitative perturbations to the model under HF?

Response: We thank the Reviewer for raising this important question. In our computer simulations, the modeling anatomy of the human SAN and SACPs was heart-specific, while cardiac electrophysiology in the RA and SAN under normal and different pathophysiological conditions were generic which were based on previous experimental or modeling studies). To simulate the electrical remodeling in the SAN complex under HF, we introduced the I_f and I_{Na} current block by 20% in the SAN complex and 5% I_{Na} block in the RA to qualitatively match experimental and modeling studies as conducted in our previous modeling study (Li et al. *Nature Communications* 2020). To simulate the impact of elevated fibrosis in HF, we used 20% fibrosis

in both SAN and SACP regions based on the previous experimental studies Lou et al. Circulation 2014 and Kalyanasundaram et al. *Circulation* 2021.

There is confusion with regard to the A1R receptor density differences within the SAN. The authors use a ratio of 10 between the center and the head/tail. However, looking at the citation provided (Fig 6c), the ratio is well less than 2.

Response: We appreciate the comment from this Reviewer. As mentioned previously, in our computer simulations, the modeling anatomy of the human SAN and SACPs was heart-specific, while cardiac electrophysiology in the RA and SAN under normal and different pathophysiological conditions were generic and qualitatively, which were based on previous experimental or modeling studies. The key information for our computer models to include is that the expression of A1R was higher in the SAN center than that in the SAN head/tail since we aimed to illustrate the mechanisms underlying A1R receptor density differences between SAN center and head/tail regardless of the absolute values of the A1R density differences within the SAN compartments as we demonstrated in Fig. 4C where we reported modelling results for the various A1R expression ratio from 10:1 to 10:9. Thus, we believe that our results and main conclusions are not affected by the exact choice of the A1R receptor density ratios within the SAN.

Reviewer #3: This looks interesting but cannot be reviewed in its current state:

Response: We thank Dr. Michael Clerx for taking the time to read and provide helpful comments on our manuscript.

1. The paper makes multiple claims to "present the first comprehensive biophysical computer model of the human SAN complex based on direct molecular, structural and functional studies in the ex-vivo human heart", but very few details of the modelling are presented (no equations, no information on how equations were chosen or parametrized, no goodness of fit, no validation through novel predictions).

Response: Please see our response to the editor with regard to the computer models used in this study. Specifically, in our computer simulations, the modeling anatomy of the human SAN and SACPs were heart-specific, while cardiac electrophysiology in the RA and SAN under normal and different pathophysiological conditions were generic which were based on previously published molecular, structural and functional studies of the human hearts.

2. In addition to discussing such matters in the paper, the manuscript should provide code and data so that it complies with PLOS comp biol's data and code sharing policies.

Response: We agree. Please see our response to the editor.
