# Supplementary material for: Mechanistic insight into the functional role of human sinoatrial node conduction pathways and pacemaker compartments heterogeneity: A computer model analysis
Source: PLoS Comput Biol. 2023 Dec 18;19(12):e1011708. doi: 10.1371/journal.pcbi.1011708 (PMC10760897; doi:10.1371/journal.pcbi.1011708)
Supplement: Attachment — Submitted filename: Response_2023-07-16_JZ.pdf [file pcbi.1011708.s003.pdf]

Reviewer's Responses to Questions

Comments to the Authors:
Please note here if the review is uploaded as an attachment.

Reviewer #1: The authors did a great job responding to my comments - I congratulate them on their interesting and substantial work.

My only remaining suggestion is to replace references 28 and 29 with more up-to-date publications by the same authors:

28. Morgan R, Colman MA, Chubb H, Seemann G, Aslanidi OV. Slow conduction in the border zones of patchy fibrosis stabilises the drivers for atrial fibrillation: Insights from multi-scale human atrial modeling. Frontiers in Physiology 2016; 7: 474.

29. Roy A, Varela M, Chubb H, MacLeod RS, Hancox JC, Schaeffter T, Aslanidi O. Identifying locations of re-entrant drivers from patient-specific distribution of fibrosis in the left atrium. PLoS Computational Biology 2020; 16: e1008086.

Response: We would like to thank Dr. Oleg Aslanidi for the positive endorsement and have updated the references as suggested.

Reviewer #2: The authors still need to be adequatelty respond to a couple of questions: 1) The intrinsic frequency gradient as assigned is not the traditional one and at odds with the recently published work of Brennan et al. The authors have not measured intrinsic frequency in any of the publications given as support. This point needs to be better discussed. Is this a prediction of the model that is unique for human SAN?

Response: We agree with this Reviewer that the intrinsic frequency needs to be discussed in the manuscript. In particular, we have provided the discussion of classical ("traditional") studies of the intrinsic frequency gradient in SAN including Opthof et al. 1987, Winslow et al. 1993, Kodama et al. 1985; 1997, Boyett et al. 1995, Boyett et al. 2000.

We would like to clarify that **the intrinsic frequency gradient assigned in our computer models was based on "the traditional one" (classical model, reviewed in Boyett et al. 2000**). The classical *gradient model*, in which there is a gradual change in the intrinsic properties of pacemaker cells from the center to periphery of the SAN, was proposed to explain how SAN could effectively pace the atria. The intrinsic pacemaker activity is greater in cells from the periphery than from the centre of the SAN (Opthof et al. 1987, Winslow et al. 1993, Kodama et al. 1985; 1997, Boyett et al. 1995). However, at the tissue level, the periphery of the SAN is connected to a large mass of atrial muscle in the crista terminalis through gap junctions resulting in the inhibition of peripheral pacemaker activity by the electrotonic influence of the highly hyperpolarized atrial muscle. On the other hand, central pacemaker cells, which are more distal from the atria are less affected by atrial electrotonic effects. Therefore, leading pacemaker activity at baseline conditions always originates in the central SAN cells, although they are intrinsically slower than the peripheral pacemaker cells.

In keeping with the gradient model, our simulated head/tail single SAN pacemaker cells ("periphery") were faster than SAN center pacemaker cells (798 ms vs 813ms SCL, Figure. 1B), which is also consistent with most experimental results (reviewed in Boyett et al. *Cardiovasc Res.* 2000). Indeed, in our experimental studies of the dog and in the human SAN *ex-vivo*, we did not measure intrinsic frequency in single pacemaker cells from different compartments of the SAN. Since, cellular modifications in our computer models were made by considering SAN regional heterogeneity data from recent human studies (Li et al. 2015; 2017; 2020; Seemann et al. 2006; Chandler et al. 2009), and from classical rabbit SAN studies by Opthof et al. 1987, Winslow et al. 1993, Kodama et al. 1985; 1997, Boyett et al. 1995 (Boyett et al. *Cardiovasc Res.* 2000). Kodama and Boyett et al. (1985; 1997) found that the intrinsic pacemaker activity of small balls of tissue (0.3 mm) taken from different regions of the SAN, is greater in balls from the periphery than in balls from the centre. This was also shown to be true by Opthof et al.1987 for small pieces of rabbit SAN pacemaker tissue.

The Reviewer referred on the rat SAN study by Brennan et al. *JACC Clin Electrophys* 2020, which reported that there were no statistically significant differences between the HRs of the intact and surgically separated superior (sSAN) and inferior SAN (iSAN) tissues. Specifically they stated that *"To examine the independent behavior of the newly identified sSAN and iSAN regions, we surgically separated the ex vivo rat SAN into 2 distinct tissues (Figure 2A). Though there were no significant differences between the HRs of the intact and separated tissues, the iSAN possessed intrinsic automaticity with a similar mean and small standard deviation as the intact preparation under baseline conditions (intact: 284.90±14.46 beats/min; sSAN: 188.10±112.30 beats/min; iSAN: 286.70±25.50 beats/min; n = 5) (Figure 2B)."* To ensure applicability of the developed human SAN models, our future computer modelling studies could test the results from Brennan et al. JACC Clin Electrophys 2020, including dissection of the SAN compartments.

As the Reviewer correctly pointed out that the prediction of our multi-scale computer SAN models is unique for human SAN. Previous studies were primarily focused on small animals, including rabbit models, which show both lateral (towards CT) and superior-inferior gradient

of intrinsic SAN pacemaker properties. However, in large animal models including canine and human SAN, the superior-inferior gradient is more prominent while lateral gradient is evident only across the SACPs due to the larger CT myocardium requiring more lateral insulation. Our previous published experimental data of the human heart *ex-vivo* (Csepe et al 2016, Li et al., *STM* 2017, *Nature Communications* 2020, *Circulation A&E* 2023) showed that the presence of the leading pacemaker was primarily in the central compartment and pacemaker shift was to inferior or superior compartments during adenosine and/ or sodium channels inhibitions. The modelling study supported these experimental observations qualitatively.

We have included the above discussions in the revised manuscript (Structural and Electrical Heterogeneity of the SAN) (Pages 15-16, Lines 389-408).

2) The space between the SAN and RA was not fully insulated but set as passive with a resting level of -62 mV. What biophysical mechanisms are in place to support this? It cannot just be assumed that since they are beside each other they will interact. Is the fat coupled electrically to the SAN and RA? What experimental proof is there for this coupling? Is this a prediction of your model? Why is this so important to the functioning of the model?
Response: Thank you for these very important questions, which have allowed us to clarify them in the manuscript as well. Please see below detailed response on all of them.

"The space between the SAN and RA was not fully insulated but set as passive with a resting level of -62 mV. What biophysical mechanisms are in place to support this?" Due to the insulating wall between the SAN and the neighbouring RA fat/fibrosis, we expected that the pacemaker depolarization in peripheral SAN region is reduced by the hyperpolarizing current flowing from the neighbouring RA, which have more negative diastolic potentials (Kirchhof et al.; Boyett et al. 2000). As described above in response to your Question #1, the cells near the center of the SAN may be subjected to less electrotonic effects due to their greater distance to the SAN border. In addition, the insulating wall will have a very minor impact on depolarization in the neighbouring RA. Therefore, in our computer modelling study, we used a value of -62.5 mV (the mean of the resting potentials of the RA and SAN cells), which has only very slight depolarisation and hyperpolarisation effects on neighbouring RA and SAN cells, respectively.

In addition, a lower diffusion coefficient (**Supplementary Table S3**) was assigned to the insulating wall to mimic the reduced gap junctions due to the regions of concomitant fibrosis and fatty infiltration (Pouliopoulos et al., *Circulation*, 2013).

"Why is this so important to the functioning of the model?" More importantly, the values we used produced realistic leading pacemakers, activation times and activation patterns compared with optical mapping results in the same heart (Li et al. *Science Translational Medicine 2017*). When we used the fully isolated boundary between the SAN and RA in the computer model, the leading SAN pacemakers always appeared along the CT border of the SAN, which was not consistent with the findings observed in our and other experimental studies (Boyett et al. *Cardiovasc Res.* 2000).

"Is the fat coupled electrically to the SAN and RA? What experimental proof is there for this coupling? Is this a prediction of your model?" Similar to others in the computer-modelling field (Sung, E. et al *Nature Cardiovascular Research*, 2022 and *Circ. Arrhythm. Electrophysiol,* 2020), the electrophysiological properties of fat–myocardium admixture (Insulation of the SAN from RA) were approximated in this study. It is known that gap junction alterations, decreased conduction velocity and abnormal electrogram signals are present in the fat–myocardium admixture, but the extent of such changes remain unknown (Sung, E. et al. *Nature Cardiovascular Research*, 2022 and *Circ. Arrhythm. Electrophysiol*, 2020).

Reviewer #3:

“Thank you for providing more details of the used model(s).
The paper is a lot clearer on the used methods now, although two major points remain to be addressed in the methods section.
1. The overal level of detail in the methods section is still not sufficient. The study should be repeatable using the information provided in this section. In addition, the very interesting results and discussion show that important work has been done, so please describe this in greater detail!”

Response: We thank the Reviewer for these positive comments. In the revised version of the manuscript, we have provided additional information to clarify our study further as suggested by the Reviewer in his detailed comments. But we would like to point out that the multi-scale computer modeling approaches have been developed in the past 20+ years (Harold and Henriquez. A computer model of normal conduction in the human atria. *Circulation Research*, 2000:87,25-36). These numerical and implementation details of human atria cell-to-organ level computer models were adequately described in previous studies, for example, Zhao et al. Circulation: Arrhythmia and Electrophysiology 2012, 5 (2), 361-370; Seemann et al. Interface focus, 2006:364 (1843); Aslanidi et al. Progress in biophysics and molecular biology, 2011, 107 (1), 156-168.

2. A particular aspect is the "projection" or "shadow" of the 3d model. This is an interesting thing to do but it is not obvious to me that there is a single, obvious way to go about it. (I see it confused R1 too). This seems to be quite a vital part of your methodology ("which is crucial for the aims of this study") so please explain the steps you took in detail, with figures and/or supplementary figures if needed.

Response: Please refer the Figure here (as the new Supplementary Figure S2). The SAN computer model was obtained using a shadow of the 3D SAN model to the XY plane (parallel to epicardium) by project all 2Ds into one plane. As a result, the 2D representation of the entire 3D human SAN structure included all SACPs and the complete SAN head/center/tail.

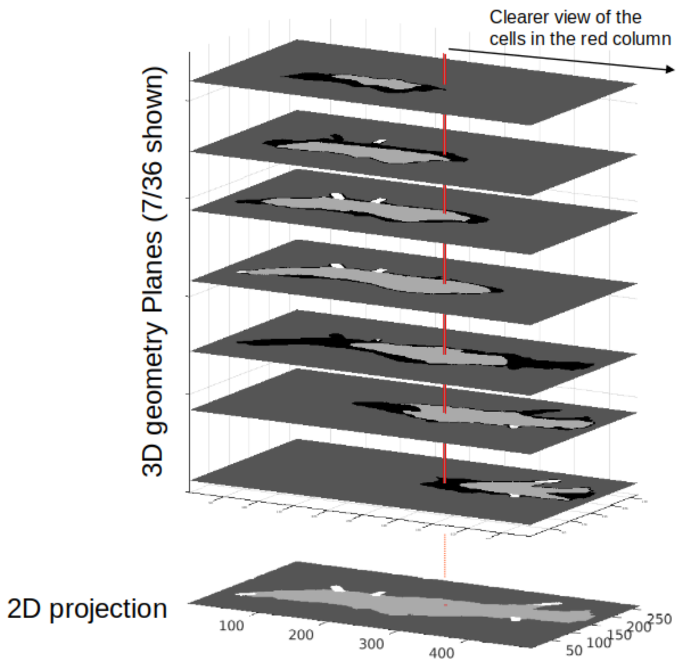

“## Detailed points
### Introduction
> which consists of ~35-50% dense connective tissue.
Could you give some hint what the rest is composed of?”
Response: The rest is composed of specialised pacemaker cells, adipose cells, immune cells and nerve fibers. This information is added to the introduction, Page 4; Lines 85-87: “*The human SAN is a single, “banana-shaped” 3D heterogeneous multicellular structure, composed of specialised pacemaker cells, adipose cells, immune cells, nerve fibers and importantly, ~35-50% dense connective tissue*”.

“> This structure is ... necessary to maintain pacemaking and conduction
Please clarify whether you mean that this hetergogeneity is known to be present, or already know to be necessary.”
Response: Yes, this heterogeneity is known to be present and is necessary.

> They established a foundation of theories
Who?
Response: These animal studies. We have revised the manuscript accordingly.

> developing new therapeutic approaches, e.g., biological pacemakers
Do you mean artificial? Either way, please provide one or more references for clarification.
Response: Biological pacemakers are an emerging field of research and technology that aims to develop alternative methods for regulating the heart's rhythm. Several approaches have been explored to develop biological pacemakers, including: Gene therapy, stem cell therapy and tissue engineering.

> control simulation studies with only a varied certain contributing factor
What do you mean by a "varied certain contributing factor"? Please rewrite.
Response: The main message here is that in experiments, it is difficult or even impossible to change just one contributing factor, for example, to separate the influence of the I_{Na} current and myofiber orientations on cardiac activation. That can be easily implemented in the computer model. We have rewritten that sentence in the revised manuscript.

### Histological imaging and reconstruction of human SAN
*> at a spatial resolution of 0.5*0.5um^2 using a 20 digital slide scanner*
Is there a word missing after 20?
Response: It should be 20X.

> High-resolutio fiber fields ... structure tensor.
Please explain in more detail or cite a reference.
Response: A reference was cited.

### Realistic human SAN computer model
In what sense is the model "realistic"? I would omit this word as it seems like quite a subjective statement to me.
Response: Removed as suggested.

Explain _how_ a "shadow" was made. Is all information from all planes projected onto the same plane? If not, how do you choose which bits to keep? How do you deal with pixels that differed only in their Z coordinate?

Response: Yes, all 2D planes were projected onto the same plane. All non-overlapping and overlapping SAN pixels become a part of the SAN mask used for computer simulations.

Does the text describe all modifications that were made? Or did you make any further changes, e.g. external concentrations set to experimental values, cell capacitances adjusted for source/sink issues, temperature changed etc? Please rewrite the text so that it's a really clear list of exactly the steps that someone would need to get the same results as you.
Response: The two cellular models we used in this study were:

- The human SAN cellular model was based on the Fabbri et al. J Physiol. 2017, which was in the CellML depository (please refer to DOI:10.36903/physiome.16550526 or Physiome repository: https://models.physiomeproject.org/workspace/648)
- The human atrial cellular model was based Ni et al.,. *Front Physiol.* 2017 (please refer to https://models.physiomeproject.org/e/807/ni_2017.cellml/view)

In our study, we applied and adapted the original cellular SAN models for different SAN regions with SAN regional heterogeneity (changed listed in the Online Tables S1&S2) to qualitatively match published experimental results (Li et al., 2017 & 2020) to investigate mechanisms of SAN automaticity and conduction, and SAN arrhythmia mechanisms under different assumptions. A custom-built C software was used to generate all computer simulations and has been described previously by our group (Zhao et al. Circulation: Arrhythmia and Electrophysiology 2012, 5 (2), 361-370) and others (Seemann and Zhang et al. 2006 https://doi.org/10.1098/rsta.2006.1781).

In our revision, custom C code and related files used for computer simulations and the human SAN model in this study have been deposited at GitHub:
https://github.com/rsha919/Human_SAN_2D_Fabbri_Ni.

Finally, we would like to point out that multi-scale computer modeling approaches have been developed in the past 20+ years (Harrild and Henriquez. A computer model of normal conduction in the human atria. *Circulation Research*, 2000:87,25-36). These numerical and implementation details of human atria cell-to-organ level computer models were adequately described in previous studies, for example, Zhao et al. Circulation: Arrhythmia and Electrophysiology 2012, 5 (2), 361-370; Seemann et al. Interface focus, 2006:364 (1843); Aslanidi et al. Progress in biophysics and molecular biology, 2011, 107 (1), 156-168.

Can you give some justification for the choice of models? I know this is a hard question and maybe quite subjective, but would be good to have a line or two on how you made this choice.
Response: The two cellular models we used in this study were the two most recent and widely used human atrial cellular models as extensive existing human studies supported this (Li et al., 2017 & 2020; Zhao et al. *JAHA* 2017).

Thank you for adding code! Can you give a bit more detail of how you got from the CellML files to your C code? Was an automated tool used? Did you find any issues that needed fixing?
Response: Yes, it has C codes under Physiome Repository and our 3D computer solver can integrate it without much further editing. There are some automatic tools to convert the codes to different computer languages under the repository.

"Did you pre-pace the system, i.e. run several beats before the ones you show in the simulations. If so, for how long? Does the system reach a "steady state" (mathematically a limit cycle) if you leave it running for e.g. 15 minutes of simulated time? How many beats were simulated before the results you show in each Figure?"

Response: For the two single-cell (SAN and atrial) models we used in the study, we used the "steady state" parameters. Due to the time-consuming nature of the multi-scale model, we did not pre-pace the system similar to most multi-scale models. Normally for our results, we will run to 10-second long (more than ten beats). 15 minutes of simulated time normally is beyond our (probably for any modellers) current capacity of the multi-scale models, given the wide arrange of the compute simulations at the cell and tissue levels.

“> It had relatively realistic geometrical loading
This is too vague and subjective: please replace this with something more precise and objective.”
Response: It was revised “*Such model reproduced the geometry of electrical connection between the SAN and the neighbouring RA and was much more efficient to run than a computer model of the SAN directly based on the 3D histological data.”*

“> We did not incorporate the SAN's internal blood vessels...focus of this study. This explains why you omitted it, but not why it was OK to do so. I'm not asking you to add this in (the whole point of a model is to simplify!) but please add something (here or refer forward to the discussion) where you give us some idea of the impact of this choice. Especially given the source/sink issues, the size of the SAN, and the crucial isolation of the SAN from the RA it seems like this is not as easily ignored as in studies of e.g. the ventricles.”
Response: The Reviewer is correct. Small blood vessels do not conduct electrical signals in the computer model, therefore, they only act as small physical barriers within the SAN. The small blood vessels may further slow down the conduction velocity within the SAN. In future, we will conduct another separate study to focus on the impact of small blood vessels on the computer simulation results.

“> and was given a constant potential between the...
Please add the exact potential, but also justify how you chose this.
I'm also interested in why you used a "neglectable" (I think the more common word here is "negligible"?) conductance instead of a zero conductance.
If, as you say, "this preset potential ... ensured a slight depolarisation and hyperpolarisation of neighboring RA and SAN cells" then by definition it wasn't negligible. Please explain what diffusivity you chose and how this affected the results.”
Response: See our response to Q2 of the Reviewer #2. In our study, we used a value of -62.5 mV, which in addition to insulation has only slight depolarisation and hyperpolarisation effects on neighbouring RA and SAN cells, respectively. It produced realistic activation patterns compared with optical mapping results. The diffusivity values were provided in the Supplementary Table S3. In the revised manuscript, we changed accordingly: *"The insulating wall (at a uniform thickness of 3 pixels) between the SAN and RA was given a constant potential of -62.5 mV, which is the mean of the resting potentials of the RA and SAN cells, and a 0.001% diffusivity of the RA diffusivity."*

“> Modifications were made by...
Please list all modifications in the supplement.
If only the ratios of conductances were adjusted, then please adapt the text to make that clear.
Like one of the other reviewers mentioned, absolute values seem more useful than ratios here.”
Response: We provided these values in the new Supplementary Table S3.

"> The SACP cell models were not able to pace themselves
Was this by design or a result of your changes? Please make a clearer distinction between (1) what you did to make your model and (2) what your model then predicted."
Response: It is a known fact that SAN cells can pace themselves, while SACP and atrial cells are not. The parameters used in Supplementary Tables S1&S2 to make sure utilised SACP cells do not pace themselves.

"> To incorporate the effects of adenosine/ACh into... concentration
I'm not sure what you mean by this. Please can you rewrite or add a one or two sentence explaination ("In brief, we...")"
Response: It is revised accordingly in the manuscript Page 7; Lines 183-185.

"> changing the density of the IKACh current as a primary effector of...
Please rewrite to clarify this. I don't think you meant to say you "change it as a primary effector"?"
Response: It is revised as *"Modelling of the relative expression of A1R in the SAN head/tail was achieved by changing the density (max conductance) of the I_{KACh} current at the head and the tail of SAN."*

"> using the same formula by Grandi et al
Please give this equation here or in the supplement (in which case you should refer to it here)."
Response: We respectively disagree. If anyone wants to replicate our results, there is no difference in seeing the replicated equation in our supplement or referring to the original reference by Grandi et al. Given that we have explicitly cited the original paper in our MS, we see no value in replicating the same formula.

"### SAN activation at baseline and...
> The developed ... model reliably reproduced
Please can you define this a bit more clearly? E.g. give real numbers etc. first before you summarise the overal performance this way; or end with a ":" and then list the reasons why you think it was reliable.
When you say "reproduced", do you mean that you made changes based on data (training), and then observed the model correctly predicting the higher-level behaviour (validation); or do you mean that you were able to tweak the model until it did (i.e. you calibrated it to the output data)? Either way this is a great achievement, but please specify it more clearly."
Response: This is just an introductory phrase and the whole paragraph below and Fig.2 explains what we mean.

"> The activation time... was about 75ms
Why "about"? What was it exactly?"
Response: It rounds to 75 ms or ~75 ms.

"> Qualitatively similar results
Please give exact numbers and allow the reader to judge this for themselves as well."
Response: Here we talk about qualitative activation patterns (shown in Figure 2): shift of pacemaker to the same direction in the model and experiment. We did not quantify it as it was for us important to reproduce just such qualitative behavior. We have provided some numbers on the activation time as requested in the next question.

"> which is slightly longer than in the model
Please give both numbers (experiment and model) in the text."
Response: It was 370 ms for the experiment versus 318 ms for the computer model.

“### The heterogeneity in expression of...
> To understand the effect of this heterogeneity on SAN function, we implemented...
Please give details in methods section and refer back to that here.”
Response: General method details are there under the Methods section.

“> we performed simulation in which the expression of A1R was...
Please add a line somewhere in the methods to say you treated expression as directly correlated with max conductance / permeability levels. This is quite uncontroversial, but still worth pointing out to e.g. experimental readers that the model does not actually include expression, translation, anchoring etc.”
Response: It is under Human SAN Computer Model under the Methods section.

“### The characteristics of SACPs dictate...
> Both INa and If currents ... were positively (or inversely) associated with the heart (or the SCL)
Had to read this twice. Maybe just write, "and inversely associated with the SCL"?”
Response: We have updated it in the revised manuscript.

“### The role of the insulation boundary...
> We found that computer models ... produced realistic activation time.
Please give exact numbers (model prediction and experimental equivalent or equivalents) so that readers can see if they agree that this was "realistic".”
Response: We have added “between 150-200 msec” in the revised manuscript.

“> a high chance of cardiac arrest
What do you mean in this case by "a high chance"? If I understand correctly it's a deterministic model, and you didn't re-run with different parameter settings etc?

In addition, we observed that cardiac arrest was induced with lower values of ACh, when compared to the insulted case.
Similarly on the next page "...increased the chance of SAN exit block", and later "had a higher tendency of exit block".”
Response: The Reviewer is correct to state that our multi-scale computer model is a deterministic model. But one has to realise that SAN dysfunction or SAN arrest/exit block is a complex disease scenery with many risk factors (ionic channels and structural components) contributing to it, it is not just 0 and 1 relationship for the individual factors. Our multi-scale computer model dissects these complex contributing factors and analyses their relative contribution. We hope this makes sense to the Reviewer.

“I would also advise to replace the term "cardiac arrest" here and in other places with "SAN arrest" as used later in the paper (there is no heart and no beating in this simulation).”
Response: We have replaced cardiac arrest by SAN arrest throughout the revised manuscript.

“### The impact of HF-induced...
> under HF remodelling (not shown).
If it's easy to do, please add this to the supplement.”
Response: The Figure is the same as shown in Figure 7B for the fibrotic remodelling in the SACPs alone (20% of fibrosis) without electrical remodelling and HF remodelling. We see no point in having two identical figures.

“### SAN is prone to...
> a train of stimuli
Please give amplitude and duration in supplement.”
Response: We applied a high voltage of 20mV for 0.0025 ms in our simulations.

“> in which we varied the degree of
Please add range over which it was varied.”
Response: Range over 0% to 30% for the I_{Na} channel block and 0% to 85% for adenosine as shown in Figure 8.

“> We found that depending on the parameters
Which ones and what values?”
Response: It refers to the I_{Na} channel block and the concentration of adenosine.

“Structural and electrical...
> However, no direct experimental data from larger species support this hypothesis.
Please clarify: no data exists at all, or existing data does not support?”
Response: This sentence was removed.

“The novelty of...
> However, much less literature exists for human SAN
Could cite Noble et al. 2012 (https://doi.org/10.1113/jphysiol.2011.224238) here, and/or the Fabbri paper (which gives a nice review in the introduction).”
Response: Here we are talking about multi-scale computer models, not single cell models. We have added “multi-scale” in the revised sentence.

“> To date, there is no... that has integrated all anatomically...
Is "all" correct here? I'm sure it doesn't integrate all data?”
Response: Our apologies and “all” is removed.

“Critical functional insights...
> made possible by the higher A1R expression
higher IKAch conductance?”
Response: Yes, higher A1R expression was modelled via higher IKAch conductance (as mentioned under Human SAN Computer Model in Method sections).

“Study limitations
> However, we do not think it...
This is not a good argument: It can still be relevant even if it's not the focus of your study.”
Response: We agree and have made the change in the revised manuscript as suggested: *“However, we suggest that taking into account 3D effects may not conceptually affect the main conclusions drawn from our study as the effects of the SAN structural and molecular features (e.g. A1R and ionic channels) on the superior/inferior shift of the leading pacemaker and preferential SACP/earliest atrial activation sites are confirmed by human SAN experiments.”*

“Conclusion
> Our novel... provides for the first time the crucial role
Other word instead of "provides"?”
Response: We have changed *"provides" to "illustrates"* in the revised manuscript as suggested.
