# Supplementary material for: Mechanistic insight into the functional role of human sinoatrial node conduction pathways and pacemaker compartments heterogeneity: A computer model analysis
Source: PLoS Comput Biol. 2023 Dec 18;19(12):e1011708. doi: 10.1371/journal.pcbi.1011708 (PMC10760897; doi:10.1371/journal.pcbi.1011708)
Supplement: Attachment — Submitted filename: Response_2023-11-09_11pm.pdf [file pcbi.1011708.s004.pdf]

Dear Dr. Zhao,

Thank you very much for submitting your manuscript "Mechanistic insight into the functional role of human sinoatrial node conduction pathways and pacemaker compartments heterogeneity: A computer model analysis" for consideration at PLOS Computational Biology. As with all papers reviewed by the journal, your manuscript was reviewed by members of the editorial board and by several independent reviewers. The reviewers appreciated the attention to an important topic. Based on the reviews, we are likely to accept this manuscript for publication, providing that you modify the manuscript according to the review recommendations.

Please prepare and submit your revised manuscript within 30 days. If you anticipate any delay, please let us know the expected resubmission date by replying to this email.

When you are ready to resubmit, please upload the following:

[1] A letter containing a detailed list of your responses to all review comments, and a description of the changes you have made in the manuscript. Please note while forming your response, if your article is accepted, you may have the opportunity to make the peer review history publicly available. The record will include editor decision letters (with reviews) and your responses to reviewer comments. If eligible, we will contact you to opt in or out
[2] Two versions of the revised manuscript: one with either highlights or tracked changes denoting where the text has been changed; the other a clean version (uploaded as the manuscript file).
Important additional instructions are given below your reviewer comments.

Thank you again for your submission to our journal. We hope that our editorial process has been constructive so far, and we welcome your feedback at any time. Please don't hesitate to contact us if you have any questions or comments.

Sincerely,

Steven A. Niederer
Guest Editor
PLOS Computational Biology

Daniel Beard
Section Editor
PLOS Computational Biology

****

Response: We thank all reviewers for taking the time to evaluate our study and provide helpful criticism and suggestions.

Reviewer #2: “The authors should comment on the inferior-superior frequency gradient which they are ignoring. Their head and tail use the same cell model. The gradient has been shown to exist in humans by Brennan et al. and has important implications for the LPS.”

Response: We appreciate the reviewer’s feedback, and want to emphasize that in our model, we didn’t ignore but accounted for the inferior-superior pacemaker gradient. In our model, we recognize SAN as a continuous pacemaker structure, with a complex and human-specific intranodal pacemaker gradients, such as center-superior and center-inferior frequency gradients.

We modelled the superior-inferior gradient in our manuscript by changing the A1R expressions in SAN head vs tail (**Figure 4D-4E**). Figure 4D shows two different scenarios of A1R expressions in SAN head vs tail: when A1R expressions is higher in the SAN tail vs head, the application of adenosine slowed SAN automaticity and shifted the leading pacemaker from SAN center (baseline condition) to head (superior). The pacemaker shift had opposite directionality (from center to tail) when SAN head and tail have the same A1R expression, despite of similar automaticity slowing (SCL from 930ms to 1229 ms vs 1134ms). We documented both scenarios in ex-vivo human donor hearts, which were studied with near-infrared transmural optical mapping and 3D histological and molecular (A1R expressions) mapping of the SAN pacemaker complex structures (Li N et. al., STM 2017, PMID: 28747516). We summarized these observations in **Figure 3B**. Thus in human hearts, the SAN automaticity gradient and superior or inferior intranodal pacemaker shift (from center to head or center to tail) depends on the heart-specific SAN compartment molecular profiles.

In the revised manuscript, we added the above clarifications to the Results sections (Lines 256-272) and further elucidate the limitations of current knowledge on superior-inferior intrinsic frequency gradient across intranodal SAN pacemaker compartments in Discussion and Study Limitations sections:

Discussion Lines 409-420: "Earlier Crick et al. (*Circulation 1994; PMID: 7908612*) reported twice higher density of parasympathetic and sympathetic nerves fibers in the SAN center vs periphery (tail). The innervation gradient may explain why sympathetic activation can shift atrial exits to superior SACP and parasympathetic activation can slow sinus rhythm and shift atrial exits inferiorly (*Fedorov et al, Circulation 2010; PMID: 20697021 and Kalyanasundaram et al., Heart Rhythm 2023; PMID: 36113768*). However, in both human and canine SAN, the direction of intranodal pacemaker shift from center to head or tail does not always correlate with the closest superior or inferior SACP *(Li et al., STM 2017; PMID: 28747516; Li et al., Circulation A&E2023; PMID: 36916270*). SAN activation can exit via the superior SACPs even though the leading pacemaker shifted inferiorly to the tail (**Figure 2B and Figure 3B**). These studies suggest that SAN automaticity gradient and superior or inferior intranodal pacemaker shift (from center to head or center to tail) depend on the heart-specific SAN compartment molecular profiles. However, no one has yet measured and compared intrinsic frequency of pacemaker cells isolated from different SAN pacemaker compartments (head, center and tail)."

Study Limitations Lines 517-519: "Due to the current paucity of human SAN compartment-specific electrophysiological data, we used the same cellular model for SAN periphery compartments (head and tail)."

For further details, the Reviewer 2 may refer to our recent comprehensive review of the human SAN mapping (Kalyanasundaram et al., Heart Rhythm 2023; PMID: 36113768) where we explained the differences between leading pacemaker sites and earliest atrial activation sites (EASs): *"… the SAN leading pacemaker location does not always correlate with the closest SACP. SAN activation can exit via the superior SACPs even though the leading pacemaker could be located in the tail and vice versa (Figure 4A-B, Left panels). EASs or SAN exit via the inferior SACP toward the IVC could be misidentified as a second "inferior SAN".*

The Reviewer 2 should also acknowledge that the choice of voltage sensitive dye determines the optical mapping ability to record activation of subsurface myocardial layers and reveal the functional continuity within intramural continuous SAN pacemaker structure:

(1) Optical mapping with conventional blue-green (excitation light) –yellow-red (emission) dyes such as di-4-ANEPPS (used in *Brennan, Efimov et al 2020* or in our human AVN mapping

paper *Hucker, Fedorov, Efimov et al. Circulation 2008; PMID: 18347223*) can record subsurface optical signals from ~1mm intramural structures, which is not sufficient to reveal the activation/conduction within the human intramural SAN and AVN structures (>1mm below Epi/Endocardial surface).

(2) Optical mapping with the near-infrared voltage-sensitive dyes such di-4-ANBDQBS (used in *Fedorov, Efimov et al JACC 2010: Li, Fedorov et al., STM 2017; PMID: 28747516*) can resolve intramural cardiac conduction up to 4 mm or even up to 10-20mm with transillumination approach (*Li, Fedorov et al., Circulation A&E2023; PMID: 36916270*). Near-infrared optical mapping could allow the optical signal to demonstrate the activation from deeper subsurface nodal structures such as human AVN or SAN (please see for details the discussion in our human AVN mapping *Fedorov, Efimov et al. Circulation A&E 2011; PMID: 21646375)*.

*Reviewer #3: "Yes similar models have been around for some time, and yes we can read about previous work in previous papers, but this does not help the reader figure out what is done in *this paper* in any level of detail. It is remarkable to me that authors from the ABI, which spearheads many efforts to combat the reproducibility crisis in computational biology and cardiac electrophysiology in particular, would make such remarks."*

Response: We would like to thank the reviewer for taking the time to read and comment on our manuscript. As we suggested before, in our manuscript we simply used and cited a formula used by Grandi et al. *Circ. Res* 2011 (PMID: 21921263; one of most widely used human atrial models) without making any changes. Additionally, in our manuscript we have provided the CellML depository links for the two used human cellular models and Tables to list changed cellular model parameters, as well as our computers codes for 3D computer models and utilized 3D human SAN anatomical models. In our view, potential readers can simply run our computer codes to replicate any results in this manuscript. Therefore, we do not understand how simply copying the same equation in our manuscript would help to combat the reproducibility crisis in computational biology and cardiac electrophysiology. On the contrary, excessive duplication could confuse potential readers about what were new in our manuscript and what is already in the existing literature.

We believe that this is consistent with current practice in our field, for instance, Bai et al. *PloS Computational Biology* 2020 (PMID: 32097431) and Bifulco et al. *Journal of the American Heart Associate* 2023 (PMID: 37581387).
